# Do Lifestyle Activities Protect Against Cognitive Decline in Aging? A Review

**DOI:** 10.3389/fnagi.2017.00381

**Published:** 2017-11-20

**Authors:** Gregory J. Christie, Tara Hamilton, Bradley D. Manor, Norman A. S. Farb, Faranak Farzan, Andrew Sixsmith, Jean-Jacques Temprado, Sylvain Moreno

**Affiliations:** ^1^Digital Health Hub, Simon Fraser University, Surrey, BC, Canada; ^2^Science and Technology for Aging Research Institute, Simon Fraser University, Surrey, BC, Canada; ^3^Institute for Aging Research, Harvard Medical School, Boston, MA, United States; ^4^Department of Psychology, University of Toronto, Mississauga, ON, Canada; ^5^School of Mechatronics and Systems Engineering, Simon Fraser University, Surrey, BC, Canada; ^6^Department of Gerontology, Simon Fraser University, Vancouver, BC, Canada; ^7^Institut des Sciences du Mouvement, Faculté des Sciences du Sport, Aix-Marseille Université, Marseille, France; ^8^School of Interactive Arts and Technology, Simon Fraser University, Surrey, BC, Canada

**Keywords:** lifestyle factors, aging, dementia, neuroplasticity, cognitive health

## Abstract

The number of patients suffering from dementia is expected to more than triple by the year 2040, and this represents a major challenge to publicly-funded healthcare systems throughout the world. One of the most effective prevention mechanisms against dementia lies in increasing brain- and cognitive-reserve capacity, which has been found to reduce the behavioral severity of dementia symptoms as neurological degeneration progresses. To date though, most of the factors known to enhance this reserve stem from largely immutable history factors, such as level of education and occupational attainment. Here, we review the potential for basic lifestyle activities, including physical exercise, meditation and musical experience, to contribute to reserve capacity and thus reduce the incidence of dementia in older adults. Relative to other therapies, these activities are low cost, are easily scalable and can be brought to market quickly and easily. Overall, although preliminary evidence is promising at the level of randomized control trials, the state of research on this topic remains underdeveloped. As a result, several important questions remain unanswered, including the amount of training required to receive any cognitive benefit from these activities and the extent to which this benefit continues following cessation. Future research directions are discussed for each lifestyle activity, as well as the potential for these and other lifestyle activities to serve as both a prophylactic and a therapeutic treatment for dementia.

## Introduction

The proportion of older adults (age 65+) is increasing steadily throughout the developed world. In the United States, the number of older adults is predicted to increase from approximately 45 million currently to 70 million by the year 2030 (Ortman et al., 2014); similarly, in the European Union the number of adults over the age of 80 is expected to grow from 5% to 12% of the population (The 2015 Ageing Report, 2015). In one noteworthy example, in 2016 Canada had, for the first time in its history, more persons over the age of 65 (16.9%) than under the age of 15 (16.6%; Government of Canada, Statistics Canada, 2016). This global trend is driven largely by the baby boom generation, born between 1946 and 1964, which began entering their senior years in 2011.

Advancing age is associated with an increased risk of developing dementia. Dementia, including Alzheimer’s disease, is a progressively degenerative disorder affecting several domains of cognitive ability. In its early, pre-clinical stage, dementia often manifests in the form of mild cognitive impairment (MCI), with deficits in cognition and memory that are greater than expected for the individual’s age but which do not yet impair that person’s ability to life independently. As symptoms progress into dementia, these impairments expand to include problems with executive functions such as planning and organizing, which limit an individual’s ability to complete basic tasks of independent living; in its later stages, dementia symptoms expand further to include additional dysfunctions in mood, personality and affect. As these symptoms worsen, patients require increasing levels of assistance with their personal care and safety. A recent estimate suggests that dementia affects approximately 24 million people globally, but given the increasing number of older adults it is projected that this number is expected to grow to approximately 81 million people by the year 2040 (World Health Organization, 2006).

This increase in dementia represents a substantial challenge to publicly-funded health care systems. Care for patients with dementia is both labor intense and long-lasting, and therefore expensive. In the United States, the combined direct and indirect care cost for dementia is estimated currently at about $236 billion per year (Alzheimer’s Association, 2016), and this value is almost certain to increase over the coming decades. In addition, current drug-based treatments have little or no efficacy in reversing or reducing the symptoms of dementia (Casey et al., 2010). Although several pharmaceutical and technological treatments are in development, these treatments face substantial research and development costs, and take a large amount of time to complete animal- and human-based clinical trials. Thus, even if viable solutions were identified, it could take years—or perhaps decades—for these solutions to be developed, validated and brought to market. This misses a crucial and rapidly closing window for prevention and early treatment.

As an alternative to conventional drug-based therapies, several groups have highlighted the potential for behavioral activities to prevent dementia (Valenzuela and Sachdev, 2006; Fratiglioni and Wang, 2007). This stems from the finding that the severity of a patient’s dementia symptoms is only weakly predicted by the degree of underlying brain pathology (for a recent review see Stern, 2009). Two non-exclusive hypotheses have been advanced to account for this finding. The first focuses on a “brain reserve capacity”, which is thought to include some combination of brain size and synapse count (Satz, 1993). For a given individual, the specific functional impairments associated with dementia would not occur (or would be weakly expressed) until damage extended beyond a critical threshold. Accordingly, a given lesion might lead to impairment in one patient with a low brain reserve capacity, but not in another patient with a higher brain reserve capacity. The second focuses on a “cognitive reserve capacity” (Stern, 2002), which proposes that the brain actively attempts to compensate for the challenge incurred by brain damage. This could include the presence of multiple neural processing networks, which would make a given cognitive process more resistant to disruption from brain damage. This could also include a compensatory mechanism, by which new neural processing networks emerge in response to brain damage.

Initially, these reserve factors were found to vary with largely immutable lifestyle factors such as education level (Sulkava et al., 1985; Zhang et al., 1990; Fratiglioni et al., 1991) and level of occupational attainment (Stern et al., 1994). Recently however, behavioral activities have shown potential in improving cognitive abilities in healthy (Scarmeas and Stern, 2003; Newson and Kemps, 2005; Hillman et al., 2008; for a review see Moreno and Farzan, 2015) and pathological populations (Särkämö et al., 2014, 2016). These benefits can induce neuroplasticity within the brain, such as increases in synaptic and dendritic receptors, and changes in neuronal growth factors (Kraft, 2012; Hötting and Röder, 2013; Moreno and Bidelman, 2014; for reviews of the neurobiology of plasticity in aging see Freret et al., 2015; Gelfo et al., 2017). A recent meta-analysis conducted by Woods et al. (2012) indicates that cognitive stimulation improves cognition in people with MCI and moderate dementia over and above any benefit from medication. Similarly, a study of same-sex twins found that participation in leisure activities during early and middle adulthood—and participation in intellectual-cultural activities in particular—was associated with a reduced risk of Alzheimer’s disease in older females (Crowe et al., 2003). This benefit, while small, is worth highlighting given the societal scale of the problem. One recent estimate is that an intervention capable of delaying the onset of dementia by just 12 months will result in a reduction of over 9 million cases of dementia (Brookmeyer et al., 2007).

Clearly, it is crucial to identify what activities and behaviors (life habits) can enhance cognitive reserve and promote healthy brain aging. A critical question, and the focus of the current review, is whether some of the most common leisure activities can fulfill this goal. We focused this review on physical exercise, meditation and musicianship, both because preliminary studies suggest that these activities may improve neurological health and because these activities are accessible and popular complementary health approaches for older adults (Clarke et al., 2015). To that end, we assess the available data indicating whether these activities are effective at reducing the incidence of dementia in our rapidly aging populations.

## Exercise

Exercise relates to activities or processes that improve one or more aspects of physical conditioning, including cardiovascular endurance, muscular strength, flexibility, balance and fine motor control. It is well known that regular physical exercise is an important part of a healthy lifestyle in all age groups. The U.S. Department of Health and Human Services recommends that adults engage in a minimum of 150 min of moderate-intensity aerobic physical activity each week (Physical Activity Guidelines Advisory Committee, 2008), but roughly 80% of adult Americans get insufficient exercise (Haskell et al., 2007). Worse still, rates of physical activity are lowest in those aged 65+ (Centers for Disease Control and Prevention (CDC), 2005). In healthy adults, regular exercise confers several well-known benefits on physical fitness and mental state, including improved health outcomes across a variety of physical conditions, better health-related quality of life, better functional capacity and better overall mental health and mood state (Penedo and Dahn, 2005). In older adults, exercise has been linked with several improvements in physical health, including reduced mortality, positive effects on lipid profiles, effective weight management, the prevention and management of Type-II diabetes, and reductions in the incidence of coronary disease, hypertension, stroke and certain cancers (Vogel et al., 2009). Physical fitness has also been shown to reduce the likelihood of falls and bone fractures in seniors, which is of particular note because older adults with dementia are at roughly three times greater risk of experiencing a fall than cognitively healthy seniors (Friedman et al., 2010). Finally, strength training has been shown to enhance quality of life in older adults by increasing their ability to accomplish everyday tasks with independence (Chou et al., 2012).

Whereas regular exercise obviously promotes physical health in seniors, it is not necessarily apparent why—or how—exercise would promote brain health. Several theories have been advanced, including that aerobic exercise promotes cerebral perfusion due to its positive effects on cardiovascular health (Rogers et al., 1990; Churchill et al., 2002; Ainslie et al., 2008); that it promotes neuro- and synaptogenesis (Kleim et al., 2002; Bugg and Head, 2011); that it increases brain-derived neurotrophic factors (Vaynman et al., 2004; Ferris et al., 2007); and that it improves insulin sensitivity and glucose control, which may reduce the incidence of amyloid plaque formation leading to Alzheimer’s disease (Watson and Craft, 2003). Other theories are more functional. For example, one recent view (Manor et al., 2010) is that a loss of physical fitness in the elderly leads to a corresponding, progressive loss in neurophysiological complexity and a corresponding reduction in information processing.

Longitudinal and randomized control trials lend initial support for the idea that physical exercise can improve cognitive health outcomes. For example, one longitudinal study followed the cognitive ability of 5925 older women over a period of 6–8 years and found that women with greater levels of physical activity were less likely to experience cognitive decline (Yaffe et al., 2001). This association could not be accounted for by differences in baseline health or confounding variables such as age, level of education, comorbid medical conditions, smoking, estrogen use, and functional limitations. In a more detailed study that assessed peak oxygen consumption as a direct measure of cardiovascular fitness, Barnes et al. (2003) evaluated physical activity from 349 individuals over the age of 55 for 6 years and also reported an inverse relationship between physical activity and level of cognitive decline.

Similarly, a pair of recent randomized controlled trial (RCT) studies suggest that cognitive benefits to older adults may be possible from relatively brief training regimes. In these studies, aerobic fitness was assessed using peak oxygen consumption before and after a 6-month intervention involving either cardiovascular exercise or stretching. Broad increases in cognitive abilities were found in participants in the exercise group but not the stretching group (Jonasson et al., 2017), and the degree of cardiovascular improvement was accompanied by increases in hippocampal volume, hippocampal functional connectivity, and enhanced connectivity between prefrontal cortex and the default mode network (Flodin et al., 2017).

Although promising, the magnitude of these benefits is typically small—for example, in the study by Barnes et al. (2003), the most- and least-fit individuals differed on a standardized test of dementia (the Mini-Mental State Exam; Folstein et al., 1975) by less than a single point out of 30 over a 6-year timespan. When existing randomized control trials are consolidated within larger meta-analyses, the benefit of exercise is more equivocal, especially for preserving cognition in healthy older adults. On the one hand, meta-analyses by Etnier et al. (1997), Colcombe and Kramer (2003), van Uffelen et al. (2008) and Smith et al. (2010) have all reported benefits, albeit typically small, from exercise on several aspects of executive functions. On the other hand, a review by Etnier et al. (2006) found that cognitive function *declined* when older adults participated in exercise regimes, and a Cochrane review by Young et al. (2015), which compared aerobic exercise programs with no intervention across 12 studies comprising 754 participants, found no significant benefit from increased fitness on any aspect of cognition in healthy older adults.

One possibility is that physical exercise may be more effective at preserving cognitive health in older adults with MCI or dementia. One recent meta-analysis, which comprised 14 RCTs of 1695 participants with MCI, reported a negligible but significant improvement in verbal fluency due to physical exercise (Gates et al., 2013). In another meta-analysis, this time comprising 11 studies and 1497 participants with MCI, aerobic exercise led to improvements in global cognitive ability and a small but significant improvement in memory (Zheng et al., 2016). In terms of physical exercise’s ability to improve cognition in older adults with dementia, an initial meta-analysis (Heyn et al., 2004) also reported moderate improvements in cognitive health. Another recent Cochrane review evaluated the effects of aerobic exercise on older adults with mild to moderate dementia (Forbes et al., 2015), and although the authors were critical of the quality of evidence available, they concluded that exercise programs may be beneficial for people with dementia in tasks of daily living.

Although there is little reason not to encourage older adults to engage in moderate physical activity, the reasons why exercise leads to heterogeneous benefits on cognitive health remain unclear, and several important questions remain unanswered on this front. Some of these questions relate to topics that are beyond the voluntary control of older adults, but which are important on a societal level. For example, it remains to be further understood how exercise interacts with the presence of the apolipoprotein E allele, which has been linked to the development of dementia. Currently, existing studies portray inconsistent patterns of results on this relationship between the benefit of exercise on cognition and the presence of this allele. On the one hand, Larson et al. (2006) found that exercise protected cognitive health in older adults irrespective of APOE genotype. In that study, the relationship between exercise and the progression of dementia-like symptoms was tracked in a longitudinal sample of 1740 men and women over the age of 65. After 6.2 years, 158 participants had developed dementia (including Alzheimer’s disease). The incidence of dementia was reduced in those who reported exercising at least three times per week, and was not affected by the presence of the APOE4 allele. Another longitudinal study (Rovio et al., 2005) assessed rates of dementia and AD in a sample of 1449 men and women over approximately 21 years. At midlife, engaging in physical activity at least twice per week was associated with a reduction in neurodegenerative disease, even after controlling for age, sex, education, follow-up time, locomotor disorders, vascular disorders, smoking and alcohol consumption. This benefit was present in all individuals regardless of apolipoprotein E genotype, but was more pronounced among those carrying the APOE ε4 allele. On the other hand, a study by Podewils et al. (2005) found that exercise did not protect cognitive health in older adults positive for the APOE4 genotype. In that study, 3375 men and women over the age of 65 participated over 5.4 years. Frequency and intensity of exercise was tracked using the 15 most popular forms of physical activity among seniors. As expected, an inverse relationship was identified between Alzheimer’s disease and energy expenditure across a number of physical activities. However, this protective factor of exercise was found only to protect those negative for the apolipoprotein E allele. Collectively, this suggests that any benefit afforded on cognitive health by exercise is potentially modulated by APOE genotype and may be insufficient to protect those who are genetically predisposed to neurodegenerative disease. A future meta-analysis that considers the mediating effects of this allele will likely provide substantial insight into this question.

Similarly, another important piece of information is the extent to which lifetime physical activity further protects against dementia. For example, Dik et al. (2003) measured the mental health of 1241 men and women between the ages of 62 and 85 years. Participants were asked about their physical exercise between the ages of 15–25 and a positive association was shown between regular physical activity in early life and level of information processing speed in older age, albeit only in men. The association was not accounted for by current physical activity or other lifestyle factors, which suggests that exercise may have a long-lasting protective benefit on cognitive health.

Other questions pertain to factors that older adults can control. For example, it remains unclear the difference—if any—between different types of exercise on cognitive health. Most studies have focused on aerobic physical exercise, although similar short-term cognitive benefits have been reported from strength/resistance training in RCT studies (Cassilhas et al., 2007; Liu-Ambrose et al., 2010, 2012). One review article (Heyn et al., 2004) reported outcome measures from multiple forms of exercise but did not systematically assess whether one type of exercise was superior to another for enhancing cognitive health. More recently, a RCT study compared aerobic training vs. resistance training in 86 older adults and found superior cognitive outcomes after 6 months for resistance training but not for aerobic training (Nagamatsu et al., 2012). Similarly, it is also possible that the cognitive benefits of exercise may be linked to the intensity of the activity. A handful of studies have reported that the intensity of physical activity, as opposed to frequency or duration, was associated with improvements in cognitive function in older adults (van Gelder et al., 2004; Angevaren et al., 2007; Brown et al., 2012). However, the meta-analysis by Smith et al. (2010) failed to identify a significant relationship between the intensity of physical activity and change in cognitive function.

In summary, although we consider the available evidence to be encouraging, additional studies are still required to more firmly establish the benefits of exercise on cognition. Several important questions remain unanswered in this field, including how type, intensity and frequency of exercise affects cognitive health; and how exercise interacts with existing cognitive health and genetic factors to affect the development and neurodegeneration of dementia. The literature would also benefit strongly from additional longitudinal studies to track the effectiveness of this intervention over longer time frames.

## Meditation

Meditation refers to a variety of techniques and practices meant to encourage mental self-regulation and the purposeful focusing of attention (Cahn and Polich, 2006; Walsh and Shapiro, 2006), often with the goal of promoting relaxation and inducing a particular state of consciousness. The specific procedure by which this mental state is attained differs between the various meditative disciplines and practitioners will often mix different techniques into their practice. This has made it extremely challenging to arrive at an operational definition of meditation for the purposes of empirical research (Cardoso et al., 2004). Divided broadly, however, three of the most popular meditative techniques emphasize *mindfulness*, *concentration* and/or *transcendence*. Respectively, these techniques correspond to focusing only on the present moment and being aware of one’s surroundings, emotions and thoughts; focusing and sustaining attention on a specific sensory stimulus or mental process; and using mantra to attain a state of relaxed awareness and minimal distractibility. Although several meditative practices have been developed within religious and/or spiritual contexts (e.g., Zen Buddhism), it appears that most—if not all—can be performed without adhering to specific beliefs, depending on the wishes of a given practitioner.

As a therapeutic technique, the concept of meditation has shifted from that of a mystic or spiritual process to one of a complementary method of healthcare delivery. In a clinical context, patients who pursue meditation frequently do so in order to improve their own physical and mental wellbeing (McCaffrey et al., 2007), and to help relieve symptoms associated with chronic disease (Nahin et al., 2012). The use of meditation (and other alternative therapies) appears to be driven not by dissatisfaction with conventional medicine but rather because patients find alternative practices to be more congruent with their own philosophical and spiritual beliefs (Astin, 1998). It is among the most popular of alternative therapies, with about 8% of American adults having engaged in some form of meditation-based treatment (Clarke et al., 2015).

Given that meditation involves creating and sustaining changes in cognitive state, it is possible that its regular practice may lead to enhanced cognitive reserve and improved neurological health in older age. Unfortunately, these beneficial effects have been difficult to ascertain due to a number of methodological issues. Whereas physical exercise has biophysical markers of exertion and fitness level, such as peak oxygen consumption, blood pressure and heart rate, no such unambiguous markers exist for meditation (although EEG-based measurements may offer potential for this). There are inherent differences in the complexity and difficulty of the various meditative techniques, which are again difficult to quantify. It is also difficult to systematically define when a user attains a meditative state, and to draw a demarcation between being in a meditative vs. a non-meditative state.

With these caveats in mind, several studies have reported enhanced cognitive abilities in cross-sectional comparisons between expert-level meditators and non-meditator controls. Some of these benefits appear to be attentional: for example, expert meditators have been found to outperform controls at tests of sustained vigilance (Valentine and Sweet, 1999; MacLean et al., 2010) and have improved perceptual stability during binocular rivalry (Carter et al., 2005), improved vigilance during sustained visual attention, and improved executive functioning and conflict resolution (Tang et al., 2007).

These attentional enhancements may also be dissociable from an improvement in working memory. One recent cross-sectional study (Prakash et al., 2010) assessed cognitive abilities from 20 males over the age of 55 who had practiced Vihangam yoga meditation for over 10 years and who had practiced daily for at least the past year. These expert meditators outperformed age- and education-matched controls on several tests of executive function, including the Stroop task, the trail-making test, the letter cancellation task and the rule shift card test. This was not the result of changes in working memory capacity, as meditators and non-meditators scored identically on a reverse digit span test. Instead, the authors concluded that meditation leads to an increase in speed of processing as well as improvements in specific cognitive domains such as set shifting and distractor inhibition.

Other studies have sought to determine whether these enhanced cognitive abilities can be developed in those without extensive experience with meditation. In one study (Slagter et al., 2007), participants were tested on a measure of visual processing speed (the attentional blink) before and after completing 3 months of training in either an intense (10–12 h/day) or mild (20 min/day for 1 week) training program in concentration-based meditation. Visual processing speed was enhanced for both groups, and was further enhanced in individuals receiving intense meditation training. Unfortunately, given the time commitment associated with the intensive meditation training, participants were not randomly assigned to groups in that study, and those undertaking the intense training were themselves already experienced meditators.

In another study, undergraduate students without meditation experience were randomly assigned to groups that received either 5 days of mindfulness-based meditation practice or 5 days of relaxation training (Tang et al., 2007). Participants in the meditation group showed greater improvement in the Attention Network Test and Profile of Mood States scale. Although this suggests that some cognitive and psychological benefit may accompany even relatively short durations of meditation training, it remains to be tested whether this benefit extends to older adults with cognitive impairment.

The long-term practice of meditation may also lead changes in the anatomical and functional architecture of the brain. Relative to control groups, expert meditators have shown enhanced gray matter thickness throughout the brain (Kang et al., 2013; Fox et al., 2014), including in regions associated with attention and sensory processing (Lazar et al., 2005), orbitofrontal cortex and the hippocampus (Luders et al., 2009), and the brainstem (Vestergaard-Poulsen et al., 2009). Expert meditators also demonstrate steady-state differences in inhibitory, slow-wave brain electrical activity (Tei et al., 2009), particularly in the frontal lobe. These differences may be involved in regulating attentional networks, allowing expert meditators to maintain attention on relevant objects and disengaging from distractors (Hasenkamp and Barsalou, 2012). Given that the frontal lobe is one of the brain regions most disrupted by aging, this again highlights the potential for meditation as a tool to preserve cognitive health in older adults.

In support of this view, recent RCT studies lend initial support for meditation as an interventional tool for people with MCI. In one study, older adults who had participated in an eight-week mindfulness-based stress reduction program showed increased functional connectivity between the posterior cingulate cortex and bilateral medial prefrontal cortex (Wells et al., 2013). Although measures of cognitive health were not reported in that study, a more recent RCT study involving 62 older adults showed improvements in memory function and corresponding improvements in resting state functional connectivity between the hippocampus and mediofrontal cortex following 12 weeks of a combined exercise-meditation training progrm (Tao et al., 2016).

Although promising, large-scale analyses portray conflicting opinions about whether meditation can meaningfully improve psychological health. One meta-analysis by Grossman et al. (2004) found evidence that meditation improves several psychological and physical dimensions, including depression, anxiety, perceived quality of life, sensory pain and level of physical impairment. More recently, another meta-analysis of 39 research reports conducted by Hofmann et al. (2010) also found that meditation-based therapy was moderately effective at improving anxiety and mood symptoms from pre- to post-treatment, regardless of number of treatment sessions. However, a separate and detailed meta-analysis (Ospina et al., 2007) was unable to draw firm conclusions on the effects of meditation practices in healthcare. In arriving at this null conclusion, the authors highlighted the experimental limitations on meditation listed previously, and characterized the existing research on the subject as suffering from poor overall methodological quality.

At present, there exist no meta-analyses on the effects of meditation on cognitive health in the elderly. Four Cochrane reviews have been performed to identify meditation’s health outcomes in adult populations. Two of these have focused on physical outcomes, with the finding that there is insufficient data available to determine the effectiveness of meditation on either hematological disease (Salhofer et al., 2016) or cardiovascular disease (Hartley et al., 2014). Two additional reviews focused on mental outcomes and were likewise unable to determine the effectiveness of meditation in treating either attention deficit hyperactivity disorder (Krisanaprakornkit et al., 2010) or anxiety disorder (Krisanaprakornkit et al., 2006).

Taken together, it is clear that the evidence linking meditation to improved cognitive health in older adults remains in the early stages. The wide number of meditative practices, as well as other challenges inherent to the quantitative assessment of this discipline, has limited both the number of extant studies and the scope of their findings. The field awaits large-scale meta-analyses before recommendations can be made, which arguably cannot be reasonably drawn until these underlying methodological issues are resolved. As a result, it is unclear what length of time is required to obtain a health benefit from meditation, nor the duration of any such benefit following cessation. Meditation may be effective at improving other aspects of psychological health, such as mood and anxiety. The spiritual/religious elements of meditation may be appealing to some users and disagreeable to others, although it should be highlighted that agnostic meditative practices exist.

## Musical Experience

Music is a highly complex sensory stimulus that is structured across several feature dimensions, including pitch, timbre, rhythm and melody. Performing music requires the integration of this complex sensory information with processes for fine motor control, working memory and performance monitoring (Zatorre and McGill, 2005; Herholz and Zatorre, 2012). Musical training has been shown to enhance neuroplasticity throughout the lifespan (Münte et al., 2002), and this has led to considerable interest in the use of musical activities as a means of improving various neurological and neurodegenerative conditions (Kraus and Chandrasekaran, 2010; Patel, 2010).

As a therapeutic intervention, many studies have investigated the neuroplastic effects of musical activities not with older adults but with children. Here, the literature has consistently revealed a clear causal link between music activities and cognitive improvements in children or young adults. Several cognitive areas have been shown to benefit from music activities, including sound processing (Tervaniemi et al., 2005), language (Moreno and Besson, 2006; Wong et al., 2007; Milovanov et al., 2008), reading (Moreno et al., 2011b), intelligence (Schellenberg and Moreno, 2010), and inhibitory control (Moreno et al., 2014). In one noteworthy study, 48 children were divided into groups that participated in either a music- or visual arts-based training program (Moreno et al., 2011a). Training for both groups consisted of two 1-h sessions each day, 5 days per week for 4 weeks. At post-training testing, only children from the music training group exhibited enhanced performance on a measure of verbal intelligence. Moreover, improvements in this intelligence score correlated with changes in functional brain plasticity during an executive function task. This demonstrated that music training can lead to transfer of a high-level cognitive skill in early childhood.

Given these benefits in children, several research groups have turned to the question of whether musical experience offers a similar cognitive benefit for older adults. Several retrospective studies have noted that individuals with extensive musical experience retain several cognitive advantages in older age. For example, trained older musicians have a superior ability to perceive speech, especially under noisy conditions (Parbery-Clark et al., 2011; Zendel and Alain, 2012) and show superior neural activity for speech perception and encoding (Parbery-Clark et al., 2012). There is also evidence pointing to a reduced loss of gray matter in trained musicians relative to musically naïve older adults (Sluming et al., 2002), which may help to offer a global reserve against dementia. Accordingly, playing a musical instrument has been found to significantly reduce the likelihood of dementia and cognitive impairment in older age (Balbag et al., 2014).

Several recent randomized control studies also suggest that relatively brief (<6 month) experiences with musical activities can improve several aspects of physical and cognitive health in older adults. For example, musical activities have been shown to improve cognitive recovery in older adults after experiencing middle cerebral artery stroke (Särkämö et al., 2008), and to improve gait dynamics and reduce the risk of falls in high-risk older adults (Trombetti et al., 2011). Notably, one study by Särkämö et al. (2014) assessed several aspects of physical and cognitive health from older adults with dementia that completed either 10 weeks of vocal training, 10 weeks of musical listening, or who received regular care. Compared with usual care, both intervention groups reported improved mood, remote episodic memory and, to lesser extents, improvements in attention, executive function and general cognition.

Finally, some of these cognitive benefits can be conferred to older adults who have not engaged in musical experiences throughout their lives. For example, executive functions were improved following 6 months of individual piano instruction for 3 h per week in older adults with no musical experience. These cognitive improvements persisted after a 3-month delay (Bugos et al., 2007; see also Van de Winckel et al., 2004), which suggests that these benefits may be long lasting. It also appears that this cognitive improvement is further enhanced with additional practice. Performance in several cognitive domains, including nonverbal memory, naming and executive processes, were enhanced in expert musicians relative to those who had trained for less than 10 years (Hanna-Pladdy and MacKay, 2011).

The benefits of music are also seemingly improved when combined with exercise, in the case of dancing. Dance combines musical experience with cardiovascular exercise, physical and motor processes and mechanisms controlling stimulus perception, emotion and memory. It also often requires complex movement sequences, which have been demonstrated to have positive effects on cognitive functions in older adults (Voelcker-Rehage et al., 2011). We have recently reviewed the utility of dance as a potential parallel to physical- and music-based therapies (Dhami et al., 2015), however the use of dance as a neuro-rehabilitative mechanism remains in its infancy.

Those studies that have assessed dance have shown promising results in older adults. For example, expert ballroom dancers were compared against age- and education-matched non-dancers and were found to have broad superiority across a range of physical and cognitive measures, notably in reaction speed, control over posture, and balance (Kattenstroth et al., 2010). These expert dancers also exhibited higher IQ, which may have mediated some of the other findings. A follow-up study (Kattenstroth et al., 2011) found further increases in physical and cognitive performance from expert, competitive dancers relative to matched expert, non-competitive dancers.

Combined physical and musical experience has also been shown to improve physical and cognitive functioning when used as an intervention. For example, a 1-year study compared cognitive functions and neuroanatomical changes between groups of healthy older adults who participated in either a dual-task which combined physical exercise with musical accompaniment, the same physical exercise without music, and a non-exercise control group. Visuospatial functioning was improved in the dual-task group, and these improvements were accompanied by preserved or enhanced brain volumes in several frontal and temporal areas (Tabei et al., 2017).

Similar benefits have also been reported at shorter time scales. In one study, sedentary older adults were assigned to either a 6-month dance course (1 h per week under a professional instructor) or a control group (Kattenstroth et al., 2013). Improvements in physical health have been observed with as little as 12 weeks of biweekly dance practice (Hui et al., 2009). Those who participated in the dance course showed significant increases in measures of physical and cognitive health, including improved attention and intelligence. Cognitive improvements may also be greater for dance than other combined therapies such as Tai Chi (Coubard et al., 2011), may be greater when participants have to rehearse specific, timed, complex dance sequences (Kimura and Hozumi, 2012) and may be more effective at preserving brain white matter than aerobic and nutritional supplementation (Burzynska et al., 2017).

Several meta-analyses have attempted to quantify the benefit of music or dance at either preventing or delaying the onset of dementia in older adults, or in managing dementia symptoms in older adults following disease onset. Two studies have shown that musical training leads to improvements in general psychological symptoms such as anxiety (Koger et al., 1999; Ueda et al., 2013), whereas another was unable to draw any useful conclusions and criticized the methodological quality of the underlying studies (Vink et al., 2003). One recent Cochrane review attempted to quantify the therapeutic value of dance as a treatment for dementia but was likewise unable to draw any useful conclusions, as that analysis was unable to identify any studies of sufficiently rigorous experimental quality for review (Karkou and Meekums, 2017). As a therapeutic solution, a recent Cochrane review concluded that musical therapy is probably effective at reducing depressive symptoms in individuals with dementia, but was unable to conclude whether cognition could be similarly improved, citing again poor-quality evidence and limited sample sizes (van der Steen et al., 2017). In summary, the neuroplastic benefits of musical training may be effective at delaying the onset of dementia in older adults, but several months or practice—and perhaps substantially more—may be required to receive the maximum protective value of this activity.

## General Discussion

The number of older adults is increasing rapidly in most developed nations, and age-related neurological health disorders, including dementia and Alzheimer’s disease, represent a substantial challenge to public health and healthcare systems. There are very few pharmacological treatments that are effective at either delaying the onset of neurodegenerative disorders or in managing the progression of symptoms. Certain life-long factors, including educational attainment and occupation, are known to contribute to brain- and cognitive-based reserves that can help offset the symptoms of dementia in later life. Although these particular factors cannot be changed by adults in later life, they do highlight that the development and progression of dementia is affected by numerous environmental factors and experiences. A critical question then, and the focus of this review, is whether popular, lifestyle-based activities can also contribute to this reserve. To that end, we evaluated the impact of lifetime experiences in physical exercise (including aerobic and resistance training), meditation (including mindfulness, concentration, yoga and tai chi practices), and musicianship (including instrument playing and dance) on brain and cognitive health in older adults. An additional priority was to determine the impact of short-term participation in these activities in later life from inexperienced older adults.

Taken together, the evidence linking lifestyle activities to a decreased risk of dementia remains equivocal. At the level of observational and RCT studies, there is encouraging evidence that moderate physical exercise and musicianship may be beneficial at improving cognitive abilities and reducing the incidence of dementia in older adults. Although this cognitive benefit is likely enhanced with lifelong exposure, a promising finding from RCT studies is that even relatively modest engagement in exercise or musical activities—roughly 5 h per week for 6 months—may help delay the effects of dementia. However, when these results are consolidated within broader meta-analyses, several confounding factors have been highlighted, including improper participant assignment to groups, insufficient blinding and relatively under-powered studies of insufficient duration. Although this tempers the overall support and recommendation of these activities, the weight of the available evidence is certainly sufficient to merit continued investigation. Furthermore, given that the risk of participation in these two activities is generally minimal, and given that exercise in particular is associated with several positive physical health outcomes, we are comfortable advocating for their use as a low-cost, scalable solution to help reduce the incidence of dementia in the growing senior population as these additional studies continue.

The evidence supporting meditation as a therapeutic tool to prevent dementia is more equivocal still. Although lifetime expertise in meditation does appear to confer several benefits for attentional processing, and may be effective at improving certain aspects of psychological health, the evidence that meditation helps protect against dementia is unclear. Confounding this are methodological issues inherent to the study of meditation, such as how to empirically measure when, and for how long, participants enter a meditative state. Recent commercial products (e.g., InterAxon’s Muse) may offer a quantitative solution to this problem by way of direct brainwave-based measurements and we encourage the adoption of more rigorous measurement techniques moving forward.

Despite the potential of these simple behaviors to promote brain health in older age there are several important, more granular questions that remain unanswered. One such question pertains to the extent to which these benefits emerge from duration of involvement. Although there is early evidence to suggest that brain health benefits from exercise and musical training can emerge from roughly 6 months of training, it is less clear if additional benefits emerge from continued practice and, importantly, how this benefit decays following cessation. This is of particular importance for physical exercise because many individuals will discontinue from an exercise regime within 1 year of commencement. Likewise, it remains to be determined which activities—or what proportion of activities—is most appropriate for enhancing healthy aging. Normal aging involves widespread structural and functional changes in neuronal, cognitive, muscular and functional systems, and our understanding of how to preserve healthy aging across these domains remains incomplete. It seems reasonable to presume that a combination of activities incorporating physical exertion, complex movement, and cognitive and social elements will be most effective at promoting healthy aging.

Another question pertains to the efficacy of these behavioral interventions in slowing the deterioration of people with MCI. Typically, accelerating memory decline begins about 7 years prior to the diagnosis of Alzheimer’s disease (Hall et al., 2001), and it is reasonable to presume that these early memory impairments may lead to reduced participation in lifestyle activities. This may result in a negative feedback loop in which memory impairment leads to loss of participation in typical lifestyle activities, leading in turn to further memory impairments. Active engagement in lifestyle activities may be effective at reducing the development of MCI, and at delaying the progression from MCI to dementia, however the trajectory of this decline remains to be quantified.

Confounding all of these problems, our knowledge of the cognitive and neural effects of these activities remains poorly understood. The aging process is associated with numerous anatomical and functional changes in the neocortex (Yang et al., 2016). Given that unambiguous behavioral benefits are themselves often difficult to observe, it is arguably unsurprising that corresponding neuroimaging studies have not provided a consistent view of how lifestyle activities affect brain structure and function. Taken collectively, the limitations of our knowledge can be largely attributed to a weak understanding of: (i) the specific cognitive effects of a given activity; (ii) individual differences in the magnitude of these cognitive effects; and (iii) how the respective brain networks mediating each cognitive ability is modified by a given activity. Two important points can be drawn from this, however. First, the effects of a given therapeutic intervention on cognition are often nuanced, and it is advisable to use a comprehensive range of neuropsychological measurements for memory, speed of processing, and executive functions (Jonasson et al., 2017). Second, the neural origins of these cognitive effects are not usually the result of circumscribed changes in brain volume but rather to enhancement of specific functional pathways, possibly involving lateral frontal cortex, the default mode network and the dorsal attention network (Franzmeier et al., 2017a,b).

As a concluding note, although the number of older adults is increasing rapidly throughout the developed world, several epidemiological studies suggest that the proportional rate of dementia may actually be decreasing over time (Christensen et al., 2013; Matthews et al., 2013; Langa, 2015). This is an encouraging finding, and it highlights the urgency to both further understand what behaviors are most effective at reducing or delaying dementia and to develop technologies and devices that can help users proactively manage their own brain health.

## Author Contributions

GJC, TH, BDN, NASF, FF, AS, J-JT and SM contributed to the development of this manuscript.

## Conflict of Interest Statement

The authors declare that the research was conducted in the absence of any commercial or financial relationships that could be construed as a potential conflict of interest.

## References

[B1] Ainslie P. N. Cotter J. D. George K. P. Lucas S. Murrell C. Shave R.. (2008). Elevation in cerebral blood flow velocity with aerobic fitness throughout healthy human ageing. J. Physiol. 586, 4005–4010. 10.1113/jphysiol.2008.158279 18635643 PMC2538930

[B2] Alzheimer’s Association. (2016). 2016 Alzheimer’s disease facts and figures. Alzheimers Dement. 12, 459–509. 10.1016/j.jalz.2016.03.001 27570871

[B3] Angevaren M. Vanhees L. Wendel-Vos W. Verhaar H. J. J. Aufdemkampe G. Aleman A.. (2007). Intensity, but not duration, of physical activities is related to cognitive function. Eur. J. Cardiovasc. Prev. Rehabil. 14, 825–830. 10.1097/HJR.0b013e3282ef995b 18043306

[B4] Astin J. A. (1998). Why patients use alternative medicine: results of a national study. JAMA 279, 1548–1553. 10.1001/jama.279.19.1548 9605899

[B5] Balbag M. A. Pedersen N. L. Gatz M. (2014). Playing a musical instrument as a protective factor against dementia and cognitive impairment: a population-based twin study. Int. J. Alzheimers Dis. 2014:e836748. 10.1155/2014/836748 25544932 PMC4269311

[B6] Barnes D. E. Yaffe K. Satariano W. A. Tager I. B. (2003). A longitudinal study of cardiorespiratory fitness and cognitive function in healthy older adults. J. Am. Geriatr. Soc. 51, 459–465. 10.1046/j.1532-5415.2003.51153.x 12657064

[B7] Brookmeyer R. Johnson E. Ziegler-Graham K. Arrighi H. M. (2007). Forecasting the global burden of Alzheimer’s disease. Alzheimers Dement. 3, 186–191. 10.1016/j.jalz.2007.04.381 19595937

[B8] Brown B. M. Peiffer J. J. Sohrabi H. R. Mondal A. Gupta V. B. Rainey-Smith S. R.. (2012). Intense physical activity is associated with cognitive performance in the elderly. Transl. Psychiatry 2:e191. 10.1038/tp.2012.118 23168991 PMC3565765

[B9] Bugg J. M. Head D. (2011). Exercise moderates age-related atrophy of the medial temporal lobe. Neurobiol. Aging 32, 506–514. 10.1016/j.neurobiolaging.2009.03.008 19386382 PMC2891908

[B10] Bugos J. A. Perlstein W. M. McCrae C. S. Brophy T. S. Bedenbaugh P. H. (2007). Individualized Piano Instruction enhances executive functioning and working memory in older adults. Aging Ment. Health 11, 464–471. 10.1080/13607860601086504 17612811

[B11] Burzynska A. Z. Jiao Y. Knecht A. M. Fanning J. Awick E. A. Chen T.. (2017). White matter integrity declined over 6-months, but dance intervention improved integrity of the fornix of older adults. Front. Aging Neurosci. 9:59. 10.3389/fnagi.2017.00059 28360853 PMC5352690

[B12] Cahn B. R. Polich J. (2006). Meditation states and traits: EEG, ERP, and neuroimaging studies. Psychol. Bull. 132, 180–211. 10.1037/0033-2909.132.2.180 16536641

[B13] Cardoso R. de Souza E. Camano L. Leite J. R. (2004). Meditation in health: an operational definition. Brain Res. Protoc. 14, 58–60. 10.1016/j.brainresprot.2004.09.002 15519952

[B14] Carter O. L. Presti D. E. Callistemon C. Ungerer Y. Liu G. B. Pettigrew J. D. (2005). Meditation alters perceptual rivalry in Tibetan Buddhist monks. Curr. Biol. 15, R412–R413. 10.1016/j.cub.2005.05.043 15936259

[B15] Casey D. A. Antimisiaris D. O’Brien J. (2010). Drugs for Alzheimer’s disease: are they effective? P T 35, 208–211. 20498822 PMC2873716

[B16] Cassilhas R. C. Viana V. A. R. Grassmann V. Santos R. T. Santos R. F. Tufik S.. (2007). The impact of resistance exercise on the cognitive function of the elderly. Med. Sci. Sports Exerc. 39, 1401–1407. 10.1249/MSS.0b013e318060111f 17762374

[B17] Centers for Disease Control and Prevention (CDC) (2005). Trends in leisure-time physical inactivity by age, sex, and race/ethnicity—United States, 1994–2004. MMWR Morb. Mort. Wkly. Rep. 54, 991–994. 16208312

[B18] Chou C.-H. Hwang C.-L. Wu Y.-T. (2012). Effect of exercise on physical function, daily living activities, and quality of life in the frail older adults: a meta-analysis. Arch. Phys. Med. Rehabil. 93, 237–244. 10.1016/j.apmr.2011.08.042 22289232

[B19] Christensen K. Thinggaard M. Oksuzyan A. Steenstrup T. Andersen-Ranberg K. Jeune B.. (2013). Physical and cognitive functioning of people older than 90 years: a comparison of two Danish cohorts born 10 years apart. Lancet 382, 1507–1513. 10.1016/s0140-6736(13)60777-1 23849796 PMC3818336

[B20] Churchill J. D. Galvez R. Colcombe S. Swain R. A. Kramer A. F. Greenough W. T. (2002). Exercise, experience and the aging brain. Neurobiol. Aging 23, 941–955. 10.1016/s0197-4580(02)00028-3 12392797

[B21] Clarke T. C. Black L. I. Stussman B. J. Barnes P. M. Nahin R. L. (2015). Trends in the use of complementary health approaches among adults: United States, 2002–2012. Natl. Health Stat. Reports 79, 1–16. 25671660 PMC4573565

[B22] Colcombe S. Kramer A. F. (2003). Fitness effects on the cognitive function of older adults a meta-analytic study. Psychol. Sci. 14, 125–130. 10.1111/1467-9280.t01-1-01430 12661673

[B23] Coubard O. A. Duretz S. Lefebvre V. Lapalus P. Ferrufino L. (2011). Practice of contemporary dance improves cognitive flexibility in aging. Front. Aging Neurosci. 3:13. 10.3389/fnagi.2011.00013 21960971 PMC3176453

[B24] Crowe M. Andel R. Pedersen N. L. Johansson B. Gatz M. (2003). Does participation in leisure activities lead to reduced risk of Alzheimer’s disease? A prospective study of swedish twins. J. Gerontol. B Psychol. Sci. Soc. Sci. 58, P249–P255. 10.1093/geronb/58.5.p249 14507930

[B25] Dhami P. Moreno S. DeSouza J. F. X. (2015). New framework for rehabilitation—fusion of cognitive and physical rehabilitation: the hope for dancing. Front. Psychol. 5:1478. 10.3389/fpsyg.2014.01478 25674066 PMC4309167

[B26] Dik M. G. Deeg D. J. H. Visser M. Jonker C. (2003). Early life physical activity and cognition at old age. J. Clin. Exp. Neuropsychol. 25, 643–653. 10.1076/jcen.25.5.643.14583 12815502

[B27] Etnier J. L. Nowell P. M. Landers D. M. Sibley B. A. (2006). A meta-regression to examine the relationship between aerobic fitness and cognitive performance. Brain Res. Rev. 52, 119–130. 10.1016/j.brainresrev.2006.01.002 16490256

[B28] Etnier J. L. Salazar W. Landers D. M. Petruzzello S. J. Han M. Nowell P. (1997). The influence of physical fitness and exercise upon cognitive functioning: a meta-analysis. J. Sport Exerc. Psychol. 19, 249–277. 10.1123/jsep.19.3.249

[B29] Ferris L. T. Williams J. S. Shen C.-L. (2007). The effect of acute exercise on serum brain-derived neurotrophic factor levels and cognitive function. Med. Sci. Sports Exerc. 39, 728–734. 10.1016/s0162-0908(08)79169-4 17414812

[B30] Flodin P. Jonasson L. S. Riklund K. Nyberg L. Boraxbekk C. J. (2017). Does aerobic exercise influence intrinsic brain activity? An aerobic exercise intervention among healthy old adults. Front. Aging Neurosci. 9:267. 10.3389/fnagi.2017.00267 28848424 PMC5554511

[B31] Folstein M. F. Folstein S. E. McHugh P. R. (1975). “Mini-mental state”. A practical method for grading the cognitive state of patients for the clinician. J. Psychiatr. Res. 12, 189–198. 10.1016/0022-3956(75)90026-6 1202204

[B32] Forbes S. C. Forbes D. Forbes S. Blake C. M. Chong L. Y. Thiessen E. J. (2015). Exercise interventions for preventing dementia or delaying cognitive decline in people with mild cognitive impairment. Cochrane Database Syst. Rev. 4:CD011706 10.1002/14651858.CD011706 PMC6378925 30383288

[B33] Fox K. C. R. Nijeboer S. Dixon M. L. Floman J. L. Ellamil M. Rumak S. P.. (2014). Is meditation associated with altered brain structure? A systematic review and meta-analysis of morphometric neuroimaging in meditation practitioners. Neurosci. Biobehav. Rev. 43, 48–73. 10.1016/j.neubiorev.2014.03.016 24705269

[B34] Franzmeier N. Göttler J. Grimmer T. Drzezga A. Áraque-Caballero M. A. Simon-Vermot L.. (2017a). Resting-state connectivity of the left frontal cortex to the default mode and dorsal attention network supports reserve in mild cognitive impairment. Front. Aging Neurosci. 9:264. 10.3389/fnagi.2017.00264 28824423 PMC5545597

[B35] Franzmeier N. Hartmann J. C. Taylor A. N. W. Araque Caballero M. Á. Simon-Vermot L. Buerger K.. (2017b). Left frontal hub connectivity during memory performance supports reserve in aging and mild cognitive impairment. J. Alzheimers Dis. 59, 1381–1392. 10.3233/JAD-170360 28731448 PMC5611800

[B37] Fratiglioni L. Grut M. Forsell Y. Viitanen M. Grafström M. Holmén K.. (1991). Prevalence of Alzheimer’s disease and other dementias in an elderly urban population: relationship with age, sex, and education. Neurology 41, 1886–1886. 10.1212/WNL.41.12.1886 1745343

[B36] Fratiglioni L. Wang H.-X. (2007). Brain reserve hypothesis in dementia. J. Alzheimers Dis. 12, 11–22. 10.3233/jad-2007-12103 17851191

[B38] Freret T. Gaudreau P. Schumann-Bard P. Billard J.-M. Popa-Wagner A. (2015). Mechanisms underlying the neuroprotective effect of brain reserve against late life depression. J. Neural Transm. 122, 55–61. 10.1007/s00702-013-1154-2 24390152

[B39] Friedman S. M. Menzies I. B. Bukata S. V. Mendelson D. A. Kates S. L. (2010). Dementia and hip fractures: development of a pathogenic framework for understanding and studying risk. Geriatr. Orthop. Surg. Rehabil. 1, 52–62. 10.1177/2151458510389463 23569663 PMC3597300

[B40] Gates N. Fiatarone Singh M. A. Sachdev P. S. Valenzuela M. (2013). The effect of exercise training on cognitive function in older adults with mild cognitive impairment: a meta-analysis of randomized controlled trials. Am. J. Geriatr. Psychiatry 21, 1086–1097. 10.1016/j.jagp.2013.02.018 23831175

[B42] Gelfo F. Mandolesi L. Serra L. Sorrentino G. Caltagirone C. (2017). The neuroprotective effects of experience on cognitive functions: evidence from animal studies on the neurobiological bases of brain reserve. Neuroscience [Epub ahead of print]. 10.1016/j.neuroscience.2017.07.065 28827089

[B43] Government of Canada, Statistics Canada (2016). Census in brief: recent trends for the population aged 15 to 64 in Canada, census year 2016. Available online at: http://www12.statcan.gc.ca/census-recensement/2016/as-sa/98-200-x/2016003/98-200-x2016003-eng.cfm. [Accessed on June 22, 2017].

[B44] Grossman P. Niemann L. Schmidt S. Walach H. (2004). Mindfulness-based stress reduction and health benefits: a meta-analysis. J. Psychosom. Res. 57, 35–43. 10.1016/S0022-3999(03)00573-7 15256293

[B45] Hall C. B. Ying J. Kuo L. Sliwinski M. Buschke H. Katz M.. (2001). Estimation of bivariate measurements having different change points, with application to cognitive ageing. Stat. Med. 20, 3695–3714. 10.1002/sim.1113 11782027

[B46] Hanna-Pladdy B. MacKay A. (2011). The relation between instrumental musical activity and cognitive aging. Neuropsychology 25, 378–386. 10.1037/a0021895 21463047 PMC4354683

[B47] Hartley L. Mavrodaris A. Flowers N. Ernst E. Rees K. (2014). Transcendental meditation for the primary prevention of cardiovascular disease. Cochrane Database Syst. Rev. 12:CD010359. 10.1002/14651858.CD010359 25436436

[B48] Hasenkamp W. Barsalou L. W. (2012). Effects of meditation experience on functional connectivity of distributed brain networks. Front. Hum. Neurosci. 6:38. 10.3389/fnhum.2012.00038 22403536 PMC3290768

[B49] Haskell W. L. Lee I.-M. Pate R. R. Powell K. E. Blair S. N. Franklin B. A.. (2007). Physical activity and public health: updated recommendation for adults from the American College of Sports Medicine and the American Heart Association. Med. Sci. Sports Exerc. 39, 1423–1434. 10.1249/mss.0b013e3180616b27 17762377

[B50] Herholz S. C. Zatorre R. J. (2012). Musical training as a framework for brain plasticity: behavior, function, and structure. Neuron 76, 486–502. 10.1016/j.neuron.2012.10.011 23141061

[B51] Heyn P. Abreu B. C. Ottenbacher K. J. (2004). The effects of exercise training on elderly persons with cognitive impairment and dementia: a meta-analysis1. Arch. Phys. Med. Rehabil. 85, 1694–1704. 10.1016/j.apmr.2004.03.019 15468033

[B52] Hillman C. H. Erickson K. I. Kramer A. F. (2008). Be smart, exercise your heart: exercise effects on brain and cognition. Nat. Rev. Neurosci. 9, 58–65. 10.1038/nrn2298 18094706

[B53] Hofmann S. G. Sawyer A. T. Witt A. A. Oh D. (2010). The effect of mindfulness-based therapy on anxiety and depression: a meta-analytic review. J. Consult. Clin. Psychol. 78, 169–183. 10.1037/a0018555 20350028 PMC2848393

[B54] Hötting K. Röder B. (2013). Beneficial effects of physical exercise on neuroplasticity and cognition. Neurosci. Biobehav. Rev. 37, 2243–2257. 10.1016/j.neubiorev.2013.04.005 23623982

[B55] Hui E. Chui B. T. Woo J. (2009). Effects of dance on physical and psychological well-being in older persons. Arch. Gerontol. Geriatr. 49, e45–e50. 10.1016/j.archger.2008.08.006 18838181

[B56] Jonasson L. S. Nyberg L. Kramer A. F. Lundquist A. Riklund K. Boraxbekk C.-J. (2017). Aerobic exercise intervention, cognitive performance, and brain structure: results from the physical influences on brain in aging (PHIBRA) study. Front. Aging Neurosci. 8:336. 10.3389/fnagi.2016.00336 28149277 PMC5241294

[B57] Kang D.-H. Jo H. J. Jung W. H. Kim S. H. Jung Y.-H. Choi C.-H.. (2013). The effect of meditation on brain structure: cortical thickness mapping and diffusion tensor imaging. Soc. Cogn. Affect. Neurosci. 8, 27–33. 10.1093/scan/nss056 22569185 PMC3541490

[B58] Karkou V. Meekums B. (2017). Dance movement therapy for dementia. Cochrane Database Syst. Rev. 2:CD011022. 10.1002/14651858.cd011022.pub2 28155990 PMC6464250

[B59] Kattenstroth J.-C. Kalisch T. Holt S. Tegenthoff M. Dinse H. R. (2013). Six months of dance intervention enhances postural, sensorimotor, and cognitive performance in elderly without affecting cardio-respiratory functions. Front. Aging Neurosci. 5:5. 10.3389/fnagi.2013.00005 23447455 PMC3581819

[B60] Kattenstroth J.-C. Kalisch T. Kolankowska I. Dinse H. R. (2011). Balance, sensorimotor, and cognitive performance in long-year expert senior ballroom dancers. J. Aging Res. 2011:e176709. 10.4061/2011/176709 21961064 PMC3179891

[B61] Kattenstroth J.-C. Kolankowska I. Kalisch T. Dinse H. R. (2010). Superior sensory, motor, and cognitive performance in elderly individuals with multi-year dancing activities. Front. Aging Neurosci. 2:31. 10.3389/fnagi.2010.00031 20725636 PMC2917240

[B62] Kimura K. Hozumi N. (2012). Investigating the acute effect of an aerobic dance exercise program on neuro-cognitive function in the elderly. Psychol. Sport Exerc. 13, 623–629. 10.1016/j.psychsport.2012.04.001

[B63] Kleim J. A. Cooper N. R. VandenBerg P. M. (2002). Exercise induces angiogenesis but does not alter movement representations within rat motor cortex. Brain Res. 934, 1–6. 10.1016/s0006-8993(02)02239-4 11937064

[B64] Koger S. M. Chapin K. Brotons M. (1999). Is music therapy an effective intervention for dementia? A meta-analytic review of literature. J. Music Ther. 36, 2–15. 10.1093/jmt/36.1.2 10519841

[B65] Kraft E. (2012). Cognitive function, physical activity, and aging: possible biological links and implications for multimodal interventions. Neuropsychol. Dev. Cogn. B Aging Neuropsychol. Cogn. 19, 248–263. 10.1080/13825585.2011.645010 22313174

[B66] Kraus N. Chandrasekaran B. (2010). Music training for the development of auditory skills. Nat. Rev. Neurosci. 11, 599–605. 10.1038/nrn2882 20648064

[B67] Krisanaprakornkit T. Krisanaprakornkit W. Piyavhatkul N. Laopaiboon M. (2006). Meditation therapy for anxiety disorders. Cochrane Database Syst. Rev. 1:CD004998. 10.1002/14651858.CD004998 16437509 PMC13418076

[B68] Krisanaprakornkit T. Ngamjarus C. Witoonchart C. Piyavhatkul N. (2010). Meditation therapies for attention-deficit/hyperactivity disorder (ADHD). Cochrane Database Syst. Rev. 6:CD006507. 10.1002/14651858.CD006507 20556767 PMC6823216

[B69] Langa K. M. (2015). Is the risk of Alzheimer’s disease and dementia declining? Alzheimers Res. Ther. 7:34. 10.1186/s13195-015-0118-1 25815064 PMC4374373

[B70] Larson E. B. Wang L. Bowen J. D. McCormick W. C. Teri L. Crane P.. (2006). Exercise is associated with reduced risk for incident dementia among persons 65 years of age and older. Ann. Intern. Med. 144, 73–81. 10.7326/0003-4819-144-2-200601170-00004 16418406

[B71] Lazar S. W. Kerr C. E. Wasserman R. H. Gray J. R. Greve D. N. Treadway M. T.. (2005). Meditation experience is associated with increased cortical thickness. Neuroreport 16, 1893–1897. 10.1097/01.WNR.0000186598.66243.19 16272874 PMC1361002

[B72] Liu-Ambrose T. Nagamatsu L. S. Graf P. Beattie B. L. Ashe M. C. Handy T. C. (2010). Resistance training and executive functions: a 12-month randomized controlled trial. Arch. Intern. Med. 170, 170–178. 10.1001/archinternmed.2009.494 20101012 PMC3448565

[B73] Liu-Ambrose T. Nagamatsu L. S. Voss M. W. Khan K. M. Handy T. C. (2012). Resistance training and functional plasticity of the aging brain: a 12-month randomized controlled trial. Neurobiol. Aging 33, 1690–1698. 10.1016/j.neurobiolaging.2011.05.010 21741129

[B74] Luders E. Toga A. W. Lepore N. Gaser C. (2009). The underlying anatomical correlates of long-term meditation: larger hippocampal and frontal volumes of gray matter. Neuroimage 45, 672–678. 10.1016/j.neuroimage.2008.12.061 19280691 PMC3184843

[B75] MacLean K. A. Ferrer E. Aichele S. R. Bridwell D. A. Zanesco A. P. Jacobs T. L.. (2010). Intensive meditation training improves perceptual discrimination and sustained attention. Psychol. Sci. 21, 829–839. 10.1177/0956797610371339 20483826 PMC3132583

[B76] Manor B. Costa M. D. Hu K. Newton E. Starobinets O. Kang H. G.. (2010). Physiological complexity and system adaptability: evidence from postural control dynamics of older adults. J. Appl. Physiol. 109, 1786–1791. 10.1152/japplphysiol.00390.2010 20947715 PMC3006415

[B77] Matthews F. E. Arthur A. Barnes L. E. Bond J. Jagger C. Robinson L.. (2013). A two-decade comparison of prevalence of dementia in individuals aged 65 years and older from three geographical areas of England: results of the Cognitive Function and Ageing Study I and II. Lancet 382, 1405–1412. 10.1016/S0140-6736(13)61570-6 23871492 PMC3906607

[B78] McCaffrey A. M. Pugh G. F. O’Connor B. B. (2007). Understanding patient preference for integrative medical care: results from patient focus groups. J. Gen. Intern. Med. 22, 1500–1505. 10.1007/s11606-007-0302-5 17846846 PMC2219802

[B79] Milovanov R. Huotilainen M. Välimäki V. Esquef P. A. A. Tervaniemi M. (2008). Musical aptitude and second language pronunciation skills in school-aged children: neural and behavioral evidence. Brain Res. 1194, 81–89. 10.1016/j.brainres.2007.11.042 18182165

[B80] Moreno S. Besson M. (2006). Musical training and language-related brain electrical activity in children. Psychophysiology 43, 287–291. 10.1111/j.1469-8986.2006.00401.x 16805867

[B83] Moreno S. Bialystok E. Barac R. Schellenberg E. G. Cepeda N. J. Chau T. (2011a). Short-term music training enhances verbal intelligence and executive function. Psychol. Sci. 22, 1425–1433. 10.1177/0956797611416999 21969312 PMC3449320

[B84] Moreno S. Friesen D. Bialystok E. (2011b). Effect of music training on promoting preliteracy skills: preliminary causal evidence. Music Percept. Interdiscip. J. 29, 165–172. 10.1525/mp.2011.29.2.165

[B81] Moreno S. Bidelman G. M. (2014). Examining neural plasticity and cognitive benefit through the unique lens of musical training. Hear. Res. 308, 84–97. 10.1016/j.heares.2013.09.012 24079993

[B82] Moreno S. Farzan F. (2015). Music training and inhibitory control: a multidimensional model. Ann. N Y Acad. Sci. 1337, 147–152. 10.1111/nyas.12674 25773629

[B85] Moreno S. Wodniecka Z. Tays W. Alain C. Bialystok E. (2014). Inhibitory control in bilinguals and musicians: event related potential (ERP) evidence for experience-specific effects. PLoS One 9:e94169. 10.1371/journal.pone.0094169 24743321 PMC3990547

[B86] Münte T. F. Altenmüller E. Jäncke L. (2002). The musician’s brain as a model of neuroplasticity. Nat. Rev. Neurosci. 3, 473–478. 10.1038/nrn843 12042882

[B87] Nagamatsu L. S. Handy T. C. Hsu C. L. Voss M. Liu-Ambrose T. (2012). Resistance training promotes cognitive and functional brain plasticity in seniors with probable mild cognitive impairment. Arch. Intern. Med. 172, 666–668. 10.1001/archinternmed.2012.379 22529236 PMC3514552

[B88] Nahin R. L. Byrd-Clark D. Stussman B. J. Kalyanaraman N. (2012). Disease severity is associated with the use of complementary medicine to treat or manage type-2 diabetes: data from the 2002 and 2007 National Health Interview Survey. BMC Complement. Altern. Med. 12:193. 10.1186/1472-6882-12-193 23088705 PMC3528411

[B89] Newson R. S. Kemps E. B. (2005). General lifestyle activities as a predictor of current cognition and cognitive change in older adults: a cross-sectional and longitudinal examination. J. Gerontol. B Psychol. Sci. Soc. Sci. 60, P113–P120. 10.1093/geronb/60.3.p113 15860780

[B90] Ortman J. M. Velkoff V. A. Hogan H. (2014). An Aging Nation: The Older Population in the United States. Washington, DC: US Census Bureau, 25–1140.

[B91] Ospina M. B. Bond K. Karkhaneh M. Tjosvold L. Vandermeer B. Liang Y.. (2007). Meditation practices for health: state of the research. Evid. Rep. Technol. Assess. (Full Rep) 155, 1–263. 17764203 PMC4780968

[B92] Parbery-Clark A. Anderson S. Hittner E. Kraus N. (2012). Musical experience offsets age-related delays in neural timing. Neurobiol. Aging 33, 1483.e1–1483.e4. 10.1016/j.neurobiolaging.2011.12.015 22227006

[B93] Parbery-Clark A. Strait D. L. Anderson S. Hittner E. Kraus N. (2011). Musical experience and the aging auditory system: implications for cognitive abilities and hearing speech in noise. PLoS One 6:e18082. 10.1371/journal.pone.0018082 21589653 PMC3092743

[B94] Patel A. D. (2010). Music, Language, and the Brain. Oxford: Oxford University Press.

[B95] Penedo F. J. Dahn J. R. (2005). Exercise and well-being: a review of mental and physical health benefits associated with physical activity. Curr. Opin. Psychiatry 18, 189–193. 10.1097/00001504-200503000-00013 16639173

[B96] Physical Activity Guidelines Advisory Committee (2008). Physical Activity Guidelines for Americans. Washington, DC: US Department of Health and Human Services, 15–34.

[B97] Podewils L. J. Guallar E. Kuller L. H. Fried L. P. Lopez O. L. Carlson M.. (2005). Physical activity, APOE genotype, and dementia risk: findings from the cardiovascular health cognition study. Am. J. Epidemiol. 161, 639–651. 10.1093/aje/kwi092 15781953

[B98] Prakash R. Dubey I. Abhishek P. Gupta S. K. Rastogi P. Siddiqui S. V. (2010). Long-term vihangam yoga meditation and scores on tests of attention. Percept. Mot. Skills 110, 1139–1148. 10.2466/04.06.22.pms.110.c.1139-1148 20866002

[B99] Rogers R. L. Meyer J. S. Mortel K. F. (1990). After reaching retirement age physical activity sustains cerebral perfusion and cognition. J. Am. Geriatr. Soc. 38, 123–128. 10.1111/j.1532-5415.1990.tb03472.x 2299115

[B100] Rovio S. Kåreholt I. Helkala E.-L. Viitanen M. Winblad B. Tuomilehto J.. (2005). Leisure-time physical activity at midlife and the risk of dementia and Alzheimer’s disease. Lancet Neurol. 4, 705–711. 10.1016/S1474-4422(05)70198-8 16239176

[B101] Salhofer I. Will A. Monsef I. Skoetz N. (2016). Meditation for adults with haematological malignancies. Cochrane Database Syst. Rev. 2:CD011157. 10.1002/14651858.cd011157.pub2 26840029 PMC7088450

[B102] Särkämö T. Altenmüller E. Rodríguez-Fornells A. Peretz I. (2016). Editorial: music, brain, and rehabilitation: emerging therapeutic applications and potential neural mechanisms. Front. Hum. Neurosci. 10:103. 10.3389/fnhum.2016.00103 27014034 PMC4783433

[B103] Särkämö T. Tervaniemi M. Laitinen S. Forsblom A. Soinila S. Mikkonen M.. (2008). Music listening enhances cognitive recovery and mood after middle cerebral artery stroke. Brain 131, 866–876. 10.1093/brain/awn013 18287122

[B104] Särkämö T. Tervaniemi M. Laitinen S. Numminen A. Kurki M. Johnson J. K.. (2014). Cognitive, emotional, and social benefits of regular musical activities in early dementia: randomized controlled study. Gerontologist 54, 634–650. 10.1093/geront/gnt100 24009169

[B105] Satz P. (1993). Brain reserve capacity on symptom onset after brain injury: a formulation and review of evidence for threshold theory. Neuropsychology 7, 273–295. 10.1037/0894-4105.7.3.273

[B106] Scarmeas N. Stern Y. (2003). Cognitive reserve and lifestyle. J. Clin. Exp. Neuropsychol. 25, 625–633. 10.1076/jcen.25.5.625.14576 12815500 PMC3024591

[B107] Schellenberg E. G. Moreno S. (2010). Music lessons, pitch processing and g. Psychol. Music 38, 209–221. 10.1177/0305735609339473

[B108] Slagter H. A. Lutz A. Greischar L. L. Francis A. D. Nieuwenhuis S. Davis J. M.. (2007). Mental training affects distribution of limited brain resources. PLoS Biol. 5:e138. 10.1371/journal.pbio.0050138 17488185 PMC1865565

[B109] Sluming V. Barrick T. Howard M. Cezayirli E. Mayes A. Roberts N. (2002). Voxel-based morphometry reveals increased gray matter density in Broca’s area in male symphony orchestra musicians. Neuroimage 17, 1613–1622. 10.1006/nimg.2002.1288 12414299

[B110] Smith P. J. Blumenthal J. A. Hoffman B. M. Cooper H. Strauman T. A. Welsh-Bohmer K.. (2010). Aerobic exercise and neurocognitive performance: a meta-analytic review of randomized controlled trials. Psychosom. Med. 72, 239–252. 10.1097/psy.0b013e3181d14633 20223924 PMC2897704

[B111] Stern Y. (2002). What is cognitive reserve? Theory and research application of the reserve concept. J. Int. Neuropsychol. Soc. 8, 448–460. 10.1017/s1355617702813248 11939702

[B112] Stern Y. (2009). Cognitive reserve. Neuropsychologia 47, 2015–2028. 10.1016/j.neuropsychologia.2009.03.004 19467352 PMC2739591

[B113] Stern Y. Gurland B. Tatemichi T. K. Tang M. X. Wilder D. Mayeux R. (1994). Influence of education and occupation on the incidence of Alzheimer’s disease. JAMA 271, 1004–1010. 10.1001/jama.271.13.1004 8139057

[B114] Sulkava R. Wikström J. Aromaa A. Raitasalo R. Lehtinen V. Lahtela K.. (1985). Prevalence of severe dementia in Finland. Neurology 35, 1025–1029. 10.1212/wnl.35.7.1025 4010941

[B115] Tabei K. Satoh M. Ogawa J. Tokita T. Nakaguchi N. Nakao K.. (2017). Physical exercise with music reduces gray and white matter loss in the frontal cortex of elderly people: the Mihama-Kiho scan project. Front. Aging Neurosci. 9:174. 10.3389/fnagi.2017.00174 28638338 PMC5461259

[B116] Tang Y.-Y. Ma Y. Wang J. Fan Y. Feng S. Lu Q.. (2007). Short-term meditation training improves attention and self-regulation. Proc. Natl. Acad. Sci. U S A 104, 17152–17156. 10.1073/pnas.0707678104 17940025 PMC2040428

[B117] Tao J. Liu J. Egorova N. Chen X. Sun S. Xue X.. (2016). Increased hippocampus-medial prefrontal cortex resting-state functional connectivity and memory function after tai chi chuan practice in elder adults. Front. Aging Neurosci. 8:25. 10.3389/fnagi.2016.00025 26909038 PMC4754402

[B118] Tei S. Faber P. L. Lehmann D. Tsujiuchi T. Kumano H. Pascual-Marqui R. D.. (2009). Meditators and non-meditators: EEG source imaging during resting. Brain Topogr. 22, 158–165. 10.1007/s10548-009-0107-4 19653090

[B119] Tervaniemi M. Just V. Koelsch S. Widmann A. Schröger E. (2005). Pitch discrimination accuracy in musicians vs nonmusicians: an event-related potential and behavioral study. Exp. Brain Res. 161, 1–10. 10.1007/s00221-004-2044-5 15551089

[B120] The 2015 Ageing Report (2015). The 2015 Ageing Report: Economic and Budgetary Projections for the 28 EU Member States (2013–2060). Luxembourg: Publications Office Available online at: http://bookshop.europa.eu/uri?target=EUB:NOTICE:KCAR15003:EN:HTML

[B121] Trombetti A. Hars M. Herrmann F. R. Kressig R. W. Ferrari S. Rizzoli R. (2011). Effect of music-based multitask training on gait, balance, and fall risk in elderly people: a randomized controlled trial. Arch. Intern. Med. 171, 525–533. 10.1001/archinternmed.2010.446 21098340

[B122] Ueda T. Suzukamo Y. Sato M. Izumi S.-I. (2013). Effects of music therapy on behavioral and psychological symptoms of dementia: a systematic review and meta-analysis. Ageing Res. Rev. 12, 628–641. 10.1016/j.arr.2013.02.003 23511664

[B124] Valentine E. R. Sweet P. L. G. (1999). Meditation and attention: a comparison of the effects of concentrative and mindfulness meditation on sustained attention. Ment. Health Relig. Cult. 2, 59–70. 10.1080/13674679908406332

[B125] Valenzuela M. J. Sachdev P. (2006). Brain reserve and dementia: a systematic review. Psychol. Med. 36, 441–454. 10.1017/s0033291705006264 16207391

[B126] Van de Winckel A. Feys H. De Weerdt W. Dom R. (2004). Cognitive and behavioural effects of music-based exercises in patients with dementia. Clin. Rehabil. 18, 253–260. 10.1191/0269215504cr750oa 15137556

[B127] van der Steen J. T. van Soest-Poortvliet M. C. van der Wouden J. C. Bruinsma M. S. Scholten R. J. Vink A. C. (2017). Music-based therapeutic interventions for people with dementia. Cochrane Database Syst. Rev. 5:CD003477. 10.1002/14651858.CD003477.pub3 28462986 PMC6481517

[B41] van Gelder B. M. Tijhuis M. A. R. Kalmijn S. Giampaoli S. Nissinen A. Kromhout D. (2004). Physical activity in relation to cognitive decline in elderly men: the FINE study. Neurology 63, 2316–2321. 10.1212/01.WNL.0000147474.29994.35 15623693

[B123] van Uffelen J. G. Z. Chin A Paw M. J. M. Hopman-Rock M. van Mechelen W. (2008). The effects of exercise on cognition in older adults with and without cognitive decline: a systematic review. Clin. J. Sport Med. 18, 486–500. 10.1097/jsm.0b013e3181845f0b 19001882

[B128] Vaynman S. Ying Z. Gomez-Pinilla F. (2004). Hippocampal BDNF mediates the efficacy of exercise on synaptic plasticity and cognition. Eur. J. Neurosci. 20, 2580–2590. 10.1111/j.1460-9568.2004.03720.x 15548201

[B129] Vestergaard-Poulsen P. van Beek M. Skewes J. Bjarkam C. R. Stubberup M. Bertelsen J.. (2009). Long-term meditation is associated with increased gray matter density in the brain stem. Neuroreport 20, 170–174. 10.1097/wnr.0b013e328320012a 19104459

[B130] Vink A. C. Bruinsma M. S. Scholten R. J. (2003). Music therapy for people with dementia. Cochrane Database Syst. Rev. 60:CD003477 10.1002/14651858.CD003477.pub2 15266489

[B131] Voelcker-Rehage C. Godde B. Staudinger U. M. (2011). Cardiovascular and coordination training differentially improve cognitive performance and neural processing in older adults. Front. Hum. Neurosci. 5:26. 10.3389/fnhum.2011.00026 21441997 PMC3062100

[B132] Vogel T. Brechat P.-H. Leprêtre P.-M. Kaltenbach G. Berthel M. Lonsdorfer J. (2009). Health benefits of physical activity in older patients: a review. Int. J. Clin. Pract. 63, 303–320. 10.1111/j.1742-1241.2008.01957.x 19196369

[B133] Walsh R. Shapiro S. L. (2006). The meeting of meditative disciplines and western psychology: a mutually enriching dialogue. Am. Psychol. 61, 227–239. 10.1037/0003-066x.61.3.227 16594839

[B134] Watson G. S. Craft S. (2003). The role of insulin resistance in the pathogenesis of Alzheimer’s disease: implications for treatment. CNS Drugs 17, 27–45. 10.2165/00023210-200317010-00003 12467491

[B135] Wells R. E. Yeh G. Y. Kerr C. Wolkin J. Davis R. B. Tan Y.. (2013). Meditation’s impact on default mode network and hippocampus in mild cognitive impairment: a pilot study. Neurosci. Lett. 556, 15–19. 10.1016/j.neulet.2013.10.001 24120430 PMC4022038

[B137] Wong P. C. M. Skoe E. Russo N. M. Dees T. Kraus N. (2007). Musical experience shapes human brainstem encoding of linguistic pitch patterns. Nat. Neurosci. 10, 420–422. 10.1038/nn1872 17351633 PMC4508274

[B138] Woods B. Aguirre E. Spector A. E. Orrell M. (2012). Cognitive stimulation to improve cognitive functioning in people with dementia. Cochrane Database Syst. Rev. 2:CD005562. 10.1002/14651858.CD005562.pub2 22336813

[B136] World Health Organization (2006). Neurological Disorders: Public Health Challenges. Geneva: World Health Organization.

[B139] Yaffe K. Barnes D. Nevitt M. Lui L.-Y. Covinsky K. (2001). A prospective study of physical activity and cognitive decline in elderly women: women who walk. Arch. Intern. Med. 161, 1703–1708. 10.1001/archinte.161.14.1703 11485502

[B140] Yang A. C. Tsai S.-J. Liu M.-E. Huang C.-C. Lin C.-P. (2016). The association of aging with white matter integrity and functional connectivity hubs. Front. Aging Neurosci. 8:143. 10.3389/fnagi.2016.00143 27378915 PMC4908139

[B141] Young J. Angevaren M. Rusted J. Tabet N. (2015). Aerobic exercise to improve cognitive function in older people without known cognitive impairment. Cochrane Database Syst. Rev. 4:CD005381. 10.1002/cca.886 25900537 PMC10554155

[B142] Zatorre R. McGill J. (2005). Music, the food of neuroscience? Nature 434, 312–315. 10.1038/434312a 15772648

[B143] Zendel B. R. Alain C. (2012). Musicians experience less age-related decline in central auditory processing. Psychol. Aging 27, 410–417. 10.1037/a0024816 21910546

[B144] Zhang M. Katzman R. Salmon D. Jin H. Cai G. Wang Z.. (1990). The prevalence of dementia and Alzheimer’s disease in Shanghai, China: impact of age, gender, and education. Ann. Neurol. 27, 428–437. 10.1002/ana.410270412 2353798

[B145] Zheng G. Xia R. Zhou W. Tao J. Chen L. (2016). Aerobic exercise ameliorates cognitive function in older adults with mild cognitive impairment: a systematic review and meta-analysis of randomised controlled trials. Br. J. Sports Med. 50, 1443–1450. 10.1136/bjsports-2015-095699 27095745

